# TV and Inactivity Are Separate Contributors to Metabolic Risk Factors in Children

**DOI:** 10.1371/journal.pmed.0030481

**Published:** 2006-12-12

**Authors:** Andrew Prentice, Susan Jebb

## Abstract

Interventions against childhood obesity will need to target both excess TV viewing and physical inactivity "separately, yet together," say Prentice and Jebb.

In 1993 the journal *Pediatrics* published an editorial entitled “TV or not TV: Fat is the question” [1]. It critiqued a study that used both cross-sectional and longitudinal analyses to ask whether after-school TV viewing was associated with adiposity and physical inactivity in adolescent American girls [2]. Surprisingly, this study found virtually no association. This counter-intuitive result emphasises the complexity of the inter-relationships between diet, physical activity, and a host of possible confounding factors in the causation of obesity and its related metabolic disturbances.

It remains possible that the obesity pandemic has unexpected causes related to infections or environmental contaminants [3], or even to the cumulative effects of assortative mating (obese people choosing obese partners, hence concentrating the genetic propensity). Nevertheless, most observers are happy to apply Occam's razor (i.e., the principle that the simplest explanation is the preferred one) and accept that obesity is the natural biological response to a massive self-imposed change in mankind's external environment, involving the ready availability of energy-dense foods and a rapid adoption of very sedentary lifestyles. However, this most basic of explanations conceals a myriad of complexities that need to be understood in order to better devise preventive and therapeutic interventions.

For eons humans and their hominid forebears have been physically active, but in the past 50 years we have discovered how to substitute fossil-fuel energy for human muscular work—from occupational work to modes of transport, and from domestic tasks to leisure pursuits. As if this were not enough, we have also invented a whole new genre of entertainment in the form of television, electronic games, and the Internet that seduces us to spend hours each day sitting almost totally inactive. Viewed from this evolutionary perspective, it becomes easy to accept that excessive TV viewing

In the past 50 years we have discovered how to substitute fossil-fuel energy for human muscular work..

and physical inactivity might be implicated as important contributory factors to the kinds of metabolic disturbances that are increasingly being reported among children in affluent societies [4]. But are they on a single causal pathway, or might they have discrete effects? In a paper in *PloS Medicine*, Ekelund et al. [5] suggest that the latter is the case.

## The European Youth Heart Study

The authors studied almost 2,000 nine- and ten-year-old and fifteen- and sixteen-year-old children from three centres (Denmark, Estonia, and Madeira) collaborating in the European Youth Heart Study. The children wore activity monitors to assess physical activity (PA) on two weekdays and two weekend days. After-school TV viewing was assessed by questionnaire, together with a range of possible confounding variables including parental social class. Adiposity was assessed by skinfold thicknesses measured at four sites and a “clustered metabolic risk” index was generated from blood pressure, fasting triglycerides, HDL cholesterol, glucose, and insulin. Two versions of this clustered metabolic risk were used; one included body fatness and one excluded it in order to be able to assess the association between adiposity and metabolic risk.

The key findings of the study are that TV viewing and PA appear to be separate entities and are independently associated with metabolic risk. TV viewing was a positive predictor of adiposity and its association with metabolic risk disappeared after adjustment for adiposity. Surprisingly, PA was not associated with adiposity but was strongly negatively associated with metabolic risk independently of adiposity and other confounding factors. In common with numerous studies in adults [e.g., 6], adiposity was positively associated with blood pressure, triglycerides, and insulin, and negatively associated with HDL cholesterol. Perhaps most surprising is the fact that these associations emerged so clearly in a relatively lean cohort of children aged only 9–15 years.

There are many strengths to this study, including its large sample size, the objective measurement of PA, and the comprehensive classification of metabolic risk. A weakness lies in the fact that TV viewing was self-reported and only covered weekday after-school viewing. The reported averages of between 1.5–1.9 hours a day are low and suggest that much of the variance in TV viewing may occur at weekends, allowing for the possibility of misclassification. This potential misclassification might obscure an inverse relationship between TV and PA, but other studies have also shown that these are not necessarily related as might at first be expected [7]. The study also gave little consideration to dietary habits, which are likely to modulate the relationships between PA and metabolic risk, both through and independently from effects on adiposity.

## Lessons for Obesity Prevention

The authors draw on related studies in the literature to propose the following tentative pathways: (1) TV viewing predicts a poor metabolic risk profile, but this likely acts through TV-associated eating and snacking and resultant effects on adiposity; and (2) PA is not reciprocally associated with duration of TV viewing and has independent effects on metabolic risks.

The public health lessons are clear. Interventions will need to target excess TV viewing and physical inactivity separately, yet together, to yield maximal improvements. Each will require a distinct set of approaches underpinned by different strategies to achieve socio-behavioural change. Reducing TV viewing requires a negative/restraining input with an inevitable element of denial, while enhancing physical activity requires positive/aspirational inputs.

The urgency of this task is underlined by a very recent publication showing that the body mass index (kg/m^2^) of Danish children aged 7–13 years is a highly significant predictor of coronary events in adulthood [8]. As the levels of paediatric obesity continue to escalate, our children are rapidly accruing personal legacies that underpin future ill-health, together with a societal debt to be inherited by future health services and the wider economy.

## Abbreviations

PA: physical activity
